# Bioinformatic prediction of epitopes in the Emy162 antigen of *Echinococcus multilocularis*

**DOI:** 10.3892/etm.2013.1142

**Published:** 2013-06-05

**Authors:** YANHUA LI, XIANFEI LIU, YUEJIE ZHU, XIAOTAO ZHOU, CHUNBAO CAO, XIAOAN HU, HAIMEI MA, HAO WEN, XIUMIN MA, JIAN-BING DING

**Affiliations:** 1 Xinjiang National Clinical Research Base of Traditional Chinese Medicine, Xinjiang Medical University, Urumqi, Xinjiang 830000;; 2 College of Basic Medicine, Xinjiang Medical University, Urumqi, Xinjiang 830011;; 3 State Key Laboratory Incubation Base of Major Diseases in Xinjiang and Xinjiang Key Laboratory of Echinococcosis, First Affiliated Hospital of Xinjiang Medical University, Urumqi, Xinjiang 830054, P. R. China

**Keywords:** Emy162, secondary structure, T cell epitopes, B cell epitopes, bioinformatics

## Abstract

The aim of the present study was to predict the secondary structure and the T- and B-cell epitopes of the *Echinococcus multilocularis* Emy162 antigen, in order to reveal the dominant epitopes of the antigen. The secondary structure of the protein was analyzed using the Gamier-Robson method, and the improved self-optimized prediction method (SOPMA) server. The T- and B-cell epitopes of Emy162 were predicted using Immune Epitope Database (IEDB), Syfpeithi, Bcepred and ABCpred online software. The characteristics of hydrophilicity, flexibility, antigenic propensity and exposed surface area were predicted. The tertiary structure of the Emy162 protein was predicted by the 3DLigandSite server. The results demonstrated that random coils and β sheets accounted for 34.64 and 21.57% of the secondary structure of the Emy162 protein, respectively. This was indicative of the presence of potential dominant antigenic epitopes in Emy162. Following bioinformatic analysis, numerous distinct antigenic epitopes of Emy162 were identified. The high-scoring T-cell epitopes were located at positions 16–29, 36–39, 97–103, 119–125 and 128–135, whilst the likely B-cell epitopes were located at positions 8–10, 19–25, 44–50, 74–81, 87–93, 104–109 and 128–136. In conclusion, five T-cell and seven B-cell dominant epitopes of the Emy162 antigen were revealed by the bioinformatic methods, which may be of use in the development of a dominant epitope vaccine.

## Introduction

*Echinococcus multilocularis* (Em) is a parasite that is a member of the order Cyclophyllidea, the class Cestoda and the phylum Platyhelminthes. Foxes act as definitive hosts in the life cycle of Em, whilst small mammals, and, in particular, rodents (e.g. mice), act as the intermediate hosts. However, humans may become infected and act as aberrant hosts through the ingestion of Em eggs. Following the oral uptake of the eggs, larvae are released from the eggs, and transported through the blood and lymph vessels to other organs, where they develop into metacestodes (also known as an alveolar hydatid). Alveolar echinococcosis (AE) (1), or alveolar hydatid disease, is a zoonotic helminthic disease caused by infection with Em larvae. AE is endemic, and is mainly confined to cold regions of high latitude or the tundra of the Northern Hemisphere, principally in the continents of North America, Europe and Asia (2). In China, AE infections are highly endemic over large areas of northwestern regions, such as Xinjiang and Gansu, as well as southwestern areas, such as the Ganzi Tibetan Autonomous Prefecture of Sichuan Province and Tibet (3,4).

Echinococcosis is severely harmful to human health, the development of the social economy and animal husbandry (5). It has a high ten-year mortality rate, and is regarded as one of the most lethal helminthic infections (6–9). The metacestode stage of Em in aberrant hosts is characterized by an alveolar structure, composed of multiple small vesicles. A typical feature of this stage is its multi-vesicular budding-like invasive growth, which leads to the infiltration of the affected organs. Alveolar hydatid disease is primarily apparent in the liver. During the initial phase, the damage to hepatic tissue that occurs as a result of alveolar hydatid infiltration includes mechanical extrusion, lipopolysaccharide (LPS) stimuli and the direct erosion of the liver. During the advanced phase, the characteristic symptoms are liver failure, hepatic coma and portal hypertension, complicated by gastrointestinal bleeding, which may be fatal (10,11). In addition to the liver, alveolar hydatid infection may be transferred through the lymphatic or blood circulation to the lungs and brain, as well as to the surface and parenchyma of other organs and the body cavity, in the manner of a malignant tumor.

The initial stages of Em larvae infection are asymptomatic, and, consequently, the diagnosis of AE only occurs incidentally, when patients undergo B-mode ultrasound screening of the liver. Therefore, AE is usually diagnosed at an advanced phase. Furthermore, in >50% of the patients who are admitted for treatment, the infection is too advanced to be treated surgically (12). At present, there are several options for the treatment of AE; surgery in combination with chemotherapy remains the first choice therapy. However, surgical resection is often incomplete, due to the diffuse infiltration of nonresectable structures. Certain procedures have been established to prevent the disease, including the regular deworming of dogs and public education; however, these preventative measures, as well as the treatment methods, are ineffective. Thus, there is a requirement to develop preventative and treatment measures that have an improved efficacy.

A previous study revealed that immunizing the intermediate hosts with an *Echinococcus granulosus (*Eg) recombinant protein vaccine protected against oncosphere infection, with an immune protective effect of up to 95–100%. Therefore, immune prevention may be an effective measure that could be taken to prevent the epidemic of hydatid disease (4). Epitopes, also known as antigenic determinants, are chemical groups that determine the specificity of the antigen. Epitopes comprise the fundamental structural subunits of the T- and B-cell antigen receptors (TCR and BCR, respectively), and the specific antibody-binding sites. Thus, epitopes may be classified as T- or B-cell epitopes (13,14). With the development of bioinformatic technology, epitope vaccines (15) have become increasingly important in immune prevention. Superior to traditional vaccines, vaccines based on epitopes are currently used as protective measures against infectious diseases. A major challenge in the development of epitope-based vaccines is to establish the immunogenic sites of the antigen that exhibit the greatest efficacy (16). Pfaff *et al* (17) demonstrated that the 146–154 amino acid (aa) and the 200–213aa peptides in the foot-and-mouth disease virus (FMDV) contained immunological epitopes for the neutralization of the virus, and that it was possible to use these epitopes for the preparation of epitope vaccines. In addition, Kouguchi *et al* (18) revealed that the Emy162 recombinant antigen induced a 74.3% protective immune effect in rats. In order to protect against the larval-stage infection of Em, Katoh *et al* (19) cloned the Em95 antigen, and generated a vaccine based on this antigen. Compared with the control group, the protection efficiency in the group immunized with the recombinant Em95 vaccine was 78.5–82.9%. These results suggested that the prevention of hydatid disease by a molecular vaccine is feasible. In the present study, we used computer technology and molecular biology software to predict and analyze the secondary and tertiary structures, and the T- and B-cell epitopes of Emy162, based on the Emy162 gene sequences. The results revealed the dominant epitopes of the Emy162 antigen, and provided experimental data for the preparation of an epitope vaccine.

## Materials and methods

### Amino acid sequence of the Emy162 protein

**Figure 1.**
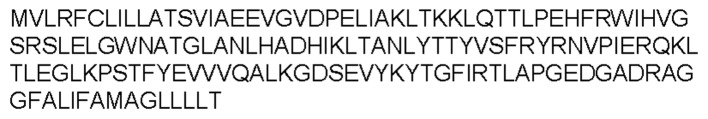
Amino acid sequence of the Emy162 protein. The protein is composed of 153 amino acid residues, and is encoded by the 5–466 region of the Emy162 mRNA.

The nucleotide sequence of Emy162 was determined using GenBank (GenBank no. AB303298.1; http://www.ncbi.nih.gov/genbank/). According to GenBank, the Emy162 protein is composed of 153 amino acid residues, encoded by the 5–466 region of the Emy162 mRNA. The amino acid sequence of the Emy162 protein is displayed in Fig. 1.

### Prediction of the secondary structure of the Emy162 protein

The secondary structure of the Emy162 protein was predicted by the improved self-optimized prediction method (SOPMA) software (http://npsa-pbil.ibcp.fr/cgi-bin/npsa_automat.pl?page=/NPSA/npsa_sopma.html) (20). The protein sequence of the Emy162 protein was input, and four conformational states, including helices, sheets, turns and coils, were analyzed. The parameters of similarity threshold and window width were set to 8 and 17, respectively, whilst the remaining parameters were not adjusted.

### Prediction of the T-cell epitopes for the Emy162 antigen

The major histocompatibility complex (MHC)-I human leukocyte antigen (HLA)-A*0201-restricted T-cell epitopes were predicted using online prediction software from the Immune Epitope Database (IEDB; http://tools.immuneepitope.org/main/index.html) (21) and Syfpeithi (http://www.syfpeithi.de). The protein sequence of the Emy162 protein was input, and the parameters were adjusted so that ‘MHC allele(s)’ was set at HLA-A*02:01, and ‘length’ was set at 8, 9 and 10. The remaining parameters were not altered.

### Prediction of the B-cell epitopes for the Emy162 antigen

The B-cell epitopes of the Emy162 protein were predicted using Bcepred (http://www.imtech.res.in/raghava/bcepred/bcepred_submission.html) and ABCpred (http://www.imtech.res.in/raghava/abcpred/) online software from the Institute of Microbial technology, India (Imtech) (22). The amino acid sequence of the Emy162 protein was input, and then the parameters were adjusted to the following values: Hydrophilicity, 2; flexibility, 1.9; exposed surface area, 2.4; and antigenic propensity, 1.8. The remaining parameters were not altered.

### Prediction of the tertiary structure of the Emy162 protein

Predictive analysis of the Emy162 protein tertiary structure was conducted using 3DLigandSite, the online ligand-binding site prediction server (http://www.sbg.bio.ic.ac.uk) (23). This web server automates the manual processes used for the prediction of ligand-binding sites in the eighth round of the critical assessment of protein structure prediction (CASP8) (24), and is a useful tool for the analysis of protein tertiary structure. The site uses ligands from similar structures to make predictions, as well as providing details of conservation information. Following the use of 3DLigandSite, the three-dimensional structure of the protein was analyzed by the Vector Alignment Search Tool (VAST; http://www.ncbi.nlm.nih.gov/Structure/VAST/vastsearch.html). The true epitopes of the structural surfaces were displayed as green and olive green spheres, whilst the white spheres represented the remainder of the protein.

## Results

### Prediction of the secondary structure of the Emy162 protein

**Figure 2.**
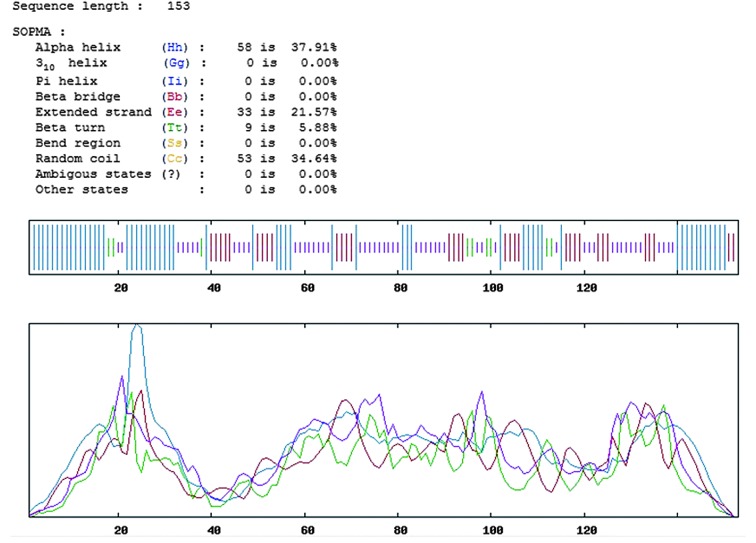
Secondary structure prediction results for the Emy162 protein. The improved self-optimized prediction method (SOPMA) software (http://npsa-pbil.ibcp.fr/cgi-bin/npsa_automat.pl?page=/NPSA/npsa_sopma.html) was used to predict the secondary structure of the Emy162 protein. An increased number of extended strands and random coils in the protein corresponded with an increased likelihood of the protein forming an antigenic epitope. Lines in different colors represent different secondary structures: Blue, α helix; green, β turn; red, extended strand; and purple, random coil.

In order to assess the antigenic features of the Emy162 protein, we predicted its secondary structure using SOPMA Server software. A greater proportion of extended strands and random coils present in the structure of the protein corresponded with an increased likelihood of the protein forming an antigenic epitope. The predicted secondary structure results for the Emy162 protein are demonstrated in Fig. 2. The results revealed that the proportion of random coils, β turns, α helices and extended strands (β folds) accounted for 34.64, 5.88, 37.91 and 21.57% of the secondary structure, respectively.

### Prediction of the T-cell epitopes for the Emy162 antigen

As mentioned previously, there are two types of epitopes: T- and B-cell epitopes. In order to develop an epitope vaccine, it is essential to determine the precise location of the epitope. In the current study, the MHC I HLA-A*0201-restricted T -cell epitopes were predicted using two online prediction software applications, IEDB and Syfpeithi, which represented the probability of a particular region forming a T-cell epitope by a score. The higher the score assigned to the region, the greater the likelihood of that region forming antigenic epitopes. The 11 regions that were revealed to score highly are listed in Table I. It is worthy of note that the two software solutions utilized different scoring systems. As predicted by the IEDB software, the high scores ranged between 87.1 and 96.05. However, the high scores predicted by the Syfpeithi software ranged between 19 and 26. Despite the difference, these high-scoring regions all had strong potential as epitope regions.

The results from the IEDB software indicated that the T-cell epitopes were located at positions 16–26, 30–39, 82–95, 97–105, 117–125 and 128–136, whereas the Syfpeithi software predicted the epitopes to be located at positions 2–19, 21–29, 36–47, 94–103, 119–135 and 141–149. The five highest-scoring regions, selected from the combined results of the two software applications, were 16–29, 36–39, 97–103, 119–125 and 128–135.

### Prediction of the B-cell epitopes for the Emy162 antigen

**Figure 3.**
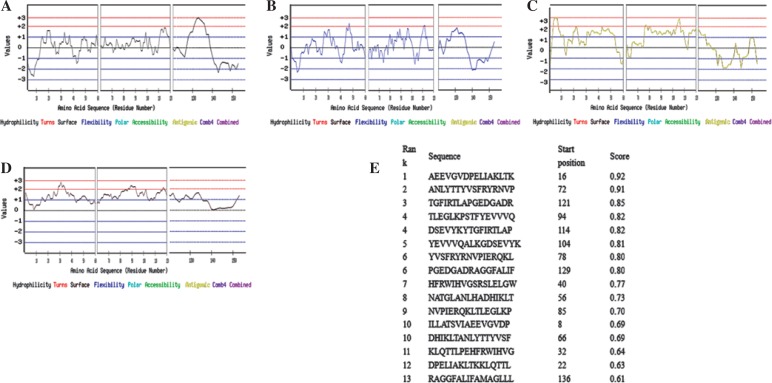
B-cell epitope prediction results for the Emy162 protein. Predictions of (A) hydrophilicity, (B) flexibility, (C) antigenic propensity, (D) exposed surface, and (E) B epitope, were determined using software from the Institute of Microbial Technology, India (Imtech) (http://www.imtech.res.in/raghava/abcpred/).

The B-cell epitopes were predicted using the Bcepred online software from Imtech. Based on the features of this software, the amino acid sequence was divided into three segments prior to the analysis, and then the parameters of hydrophilicity, flexibility, the antigenic propensity and the exposed surface area of antigen were analyzed. The results are displayed in Fig. 3. The region that was revealed to have high hydrophilicity was 128–139 (amino acid sequence: APGEDGADRAGG) (Fig. 3A), whilst the flexible regions were 44–55 (IHVGSRS) and 109–115 (QALKGDS; Fig. 3B). The possible antigenic regions were demonstrated to be 1–10 (MVLRFCLILL), 19–25 (VGVDPEL), 74–81 (LYTTYVSF) and 101–109 (STFYEVVVQ; Fig. 3C). The exposed surface areas were located at positions 28–36 (KLTKKLQTT) and 87–93 (PIERQKL; Fig. 3D).

To further verify these results, the prediction was also conducted using an additional online software application, ABCpred. As indicated in Fig. 3E, the regions with high scores were 16–31, 40–54, 56–70, 72–100, 104–112 and 114–136.

A combination of the results predicted by the different methods indicated that the potential B-cell epitopes of the Emy162 antigen were located at positions 8–10, 19–25, 44–50, 74–81, 87–93, 104–109 and 128–136.

### Prediction of the tertiary structure of the Emy162 protein

**Figure 4.**
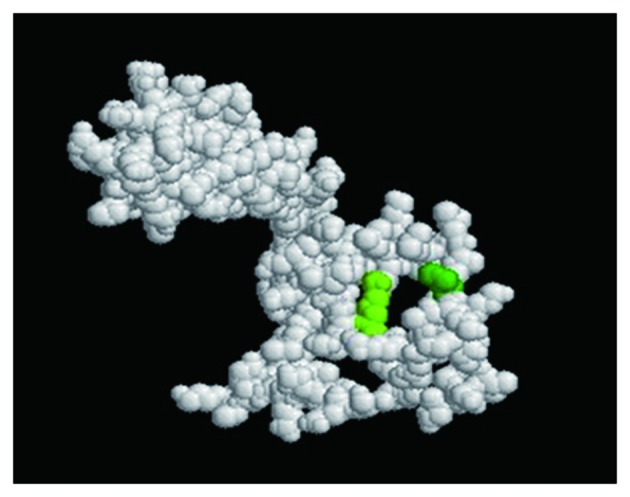
Tertiary structure prediction results for the Emy162 protein. This image was created using 3DLigandSite (http://www.sbg.bio.ic.ac.uk). The green and olive green spheres represent the potential epitopes, with the green spheres indicating stronger potential. The white spheres represent the remainder of the protein.

The tertiary structure of the Emy162 protein was obtained using the 3DLigandSite software, and compared with the structure from VAST. The results of the predicted conformations of the epitopes are displayed in Fig. 4. The olive green and the green spheres indicate potential epitopes.

## Discussion

The research and development of epitope vaccines is a difficult and highly targeted technology, which comprehensively utilizes molecular biology and immunology. A key step in the preparation of the vaccines is obtaining the necessary information concerning the epitope. In recent years, with the development of bioinformatics, epitope prediction has improved in simplicity and significance. Performing predictions with a multi-parameter and -method analysis greatly enhances the accuracy of the epitope prediction. In a study by Shen *et al* (25) the secondary structure and surface characteristics of the follicle stimulating hormone receptor (FSHR) extracellular domain were analyzed by DNAStar Protean software, and the B-cell epitope prediction was conducted using alternative online software. The possible antigenic epitopes of the FSHR extracellular domain were predicted, the peptides of the epitopes were synthesized and then the immunogenicity of these peptides was determined. In a different study, conducted by Li *et al* (26), the B-cell dominant epitopes of the Epstein-Barr nuclear antigen (EBNA)-1 were predicted, based on its gene sequences, using SOPMA, as well as the Garnier-Osguthorpe-Robson (GOR) and hierarchical neural network (HNN) methods. In addition, the transmembrane domain and various parameters, including hydrophilicity, were analyzed. Following this, the sequence alignment of the predicted EBNA-1 B-cell epitopes and the sequences of interrelated human autoantigens were contrastively analyzed using blastp. The secondary structure of a protein is closely correlated with its epitope distribution. Hydrophilicity and antigenicity are the primary factors involved in epitope formation, although interrelated factors, such as flexibility, the exposed surface area and the conformation of the secondary structure, are also important. Thus, we analyzed the secondary structure of the Emy162 protein in order to obtain the antigenic features of the protein. However, as the combined effects of T- and B-cells are necessary for the elimination of antigens, it was also important to analyze the T- and B-cell epitopes of the Emy162 antigen. Therefore, we predicted the T- and B-cell epitopes using a multi-parameter and -method analysis. A comprehensive analysis of this nature may, in the future, improve the accuracy and specificity of epitope prediction.

The α helices and β sheets, in the secondary structure of proteins, are very regular structures, and are not readily deformed. This is due to the presence of hydrogen bonds, which act to maintain structural stability. However, α helices and β sheets are usually located inside the protein, which is difficult for ligand binding. By contrast, the β turn and the random coil regions are located on the surface of the protein, where it is necessary for the surface structure to make appropriate changes to meet the functional needs of the protein. Therefore, these structures are suitable for binding ligands, and have a high possibility of forming epitopes. As analyzed by SOPMA Server software, the proportions of α helices and β sheets were 37.91 and 21.57%, respectively. This result indicates that the Emy162 antigen had a good stability. Random coils and β turns, which represented the potential epitope regions, accounted for 34.64 and 5.88% of the protein, respectively. Collectively, there were eight potential epitope regions in the Emy162 antigen, and, among these eight, there were high proportions of random coils located at positions 17–28, 61–72, 102–108 and 138–145. This suggested that these four regions had stronger antigenicity.

The accuracy rate of the MHC I epitope prediction in the prediction of T-cell epitopes has been demonstrated to be up to 90% (27). In the Chinese population, HLA-A*0201 is the most common HLA-I molecule, with a positive rate of 55%. Therefore, in the present study, the HLA-A*0201-restricted epitopes of the Emy162 antigen were analyzed using the IEDB and Syfpeithi online software applications. The T-cell epitopes of the Emy162 antigen were predicted to be located at positions 16–29, 36–39, 97–103, 119–125 and 128–135, as these regions exhibited high scores.

In order to improve the accuracy of the B-cell epitope prediction for the Emy162 antigen, a multi-method and -parameter analysis was utilized. The hydrophilicity parameter prediction reflected the position of a hydrophilic residue in the entire amino acid sequence of the antigen. The amino acid residues of the protein may be divided into two types: hydrophilic and hydrophobic residues. Hydrophobic residues are, in general, packaged inside the protein, whereas hydrophilic residues are located on the surface of the protein. This conformation is favorable for the binding of hydrophilic residues to polar molecules in the solution. Such binding neutralizes the charge of the protein, and enables the protein to maintain its state of free minimal energy. Thus, the hydrophilic regions are closely associated with the epitopes (28). The flexibility parameter prediction indicated the ability of the protein to bend and fold. With an increased degree of flexibility, the polypeptide skeleton of the protein has an improved capacity to fold and bend, thus facilitating the formation of the secondary structure (29). The antigenic propensity analysis demonstrated the immunogenic regions of the antigen. Potentially dominant epitopes are particularly likely to be located in regions with a high antigenic propensity. The exposed surface area analysis reflected the distribution of the residues in the outer layer of the protein (30). The exposed surface areas of the antigen have an enhanced probability of coming into contact with the solvent molecules. In combination with these parameters, two online software applications were used to predict the B cell epitopes. Seven potential epitope regions were revealed, located at positions 8–10, 19–25, 44–50, 74–81, 87–93, 104–109 and 128–136.

Protein tertiary structure, one of the higher-order structures of the protein, is a three-dimensional conformation of the naturally folded protein. Tertiary structure has a globular conformation that is formed by the further coiling and folding of the secondary structure. Therefore, the prediction of the tertiary structure was a useful supplement to the prediction of the Emy162 antigenic epitopes.

The aim of this study was to obtain the bioinformatic characteristics of the Emy162 antigen. SOPMA Server software and the 3DLigandsite were used to predict the secondary and tertiary structures of the Emy162 antigen, respectively, whilst a number of online prediction software applications, including IEDB, Syfpeithi, Bcepred and ABCpred, were used for the T- and B-cell epitope predictions. The prediction results for the secondary and tertiary structures suggested that there were potential epitopes present in the Emy162 antigen. The T- and B-cell epitope prediction results indicated that the T-cell epitopes were located at positions 16–29, 36–39, 97–103, 119–125 and 128–135, whereas the B-cell epitopes were located at positions 8–10, 19–25, 44–50, 74–81, 87–93, 104–109 and 128–136. These regions were the potential dominant epitopes of the Emy162 antigen. The results of our study provide experimental data for the identification and screening of epitopes, and may be used for the development of epitope vaccines that have an enhanced safety and efficacy. This may result in the provision of improved regimens for the prevention and treatment of AE.

## Figures and Tables

**Table I.** Analysis of the MHC I HLA-A*0201-restricted T-cell epitopes using IEDB and Syfpeithi online prediction software.

No.	IEDB			Syfpeithi		
	
	Starting point	Amino acid sequence	Score	Starting point	Amino acid sequence	Score
1	16	AEEVGVDPE	96.05	39	LIAKLTKKL	26
2	23	PELIAKLTK	87.30	141	ALIFAMAGL	26
3	26	IAKLTKKLQ	93.00	6	CLILIATSV	25
4	30	TKKLQTTLP	90.45	2	VLRFCLILI	24
5	31	KKLQTTLPE	92.45	7	LILIATSVI	22
6	82	RYRNVPIER	90.20	21	VDPELIAKL	22
7	84	RNVPIERQK	95.30	13	SVIAEEVGV	21
8	88	IERQKLTLE	87.10	126	TLAPGEDGA	21
9	97	GLKPSTFYE	89.10	36	TLPEHFRWI	20
10	117	VYKYTGFIR	91.95	94	TLEGLKPST	19
11	128	APGEDGADR	87.90	119	KYTGFIRTL	19

IEDB: http://tools.immuneepitope.org/main/index/html; Syfpeithi: http://www.syfpeithi.de.
